# Rethinking cancer evolution: from genetic mutations to complex information systems in tumor reversion

**DOI:** 10.1093/emph/eoae032

**Published:** 2024-12-13

**Authors:** Mesut Tez

**Affiliations:** Department of Surgery, University of Health Sciences, Ankara City Hospital, Cankaya, Ankara, Turkey

**Keywords:** cancer, evolution, genetic, mutations, information systems

## Abstract

Cancer research has historically focused on the somatic mutation theory, viewing cancer as a consequence of genetic mutations. However, this perspective has limitations in explaining phenomena like tumor reversion and cancer heterogeneity. This paper introduces an alternative approach: viewing cancer as a complex information-processing system shaped by its microenvironment. By integrating historical data on tumor reversion and insights into evolutionary dynamics, I propose a reframing of cancer biology. This process-oriented perspective highlights the role of cellular plasticity and adaptive behaviors, offering new pathways for therapeutic development.

## INTRODUCTION

Cancer is perhaps one of the most significant public health problems today. Over the last 40 years, the survival rate of patients suffering from various types of cancer has increased by up to 30% [1]. This is an appreciable result, though it has largely been due to early diagnosis and the development of improved surgical and radiation-based therapies, while medical treatments have provided only marginal benefits in solid cancers [1]. A similar trend is emerging regarding target-based drugs, which show limited curative potential [2].

While the somatic mutation theory (SMT) has dominated cancer research, it has failed to fully account for critical phenomena such as tumor heterogeneity and spontaneous regression. According to this gene-centric/reductionist theory, cancer results from the accumulation of gene mutations that begin in a single cell and are passed down through successive generations via clonal expansion. The fundamental assumptions of the theory are based on the notion that gene mutations are the molecular basis of cancer [3]. There are several reasons why this model has gained popularity. It highlights the power of using a theoretical framework to understand cancer. By integrating genetics (gene mutations) with evolution (stepwise accumulation) and cancer phenotypes, it simplifies the understanding of cancer. The simplicity of the model has driven researchers to search for early markers and molecular therapeutic targets. It also aligns well with biological processes and the idea that small changes over time lead to significant shifts. It is expected that the stepwise pattern will become clear as more data accumulate, despite the current heterogeneity seen in clinical data [3].

However, some contradictory observations and questions have arisen against this theory. The numerous gene mutations detected in cancer have been classified as “driver” and “passenger” mutations. Driver mutations are thought to provide a fitness advantage that can lead to clonal expansion, while passenger mutations are assumed to have no impact on cancer progression. However, many cancer genome analyses have found fewer driver mutations than expected, and some normal tissues even harbor typical cancer driver mutations. This challenges the traditional cancer gene mutation theory. To explain the scarcity of driver mutations, the “mini-driver” model has been proposed, suggesting that many mutations each contribute small effects to overall cancer fitness. Additionally, the roles of driver and passenger mutations may change during different phases of cancer evolution, depending on environmental conditions. The interaction and competition between driver and passenger mutations are still not fully understood, and it remains unclear how these dynamics, along with karyotype changes, influence cancer progression at the gene level [3, 4].

The growth process and genomic patterns of human cancer are difficult to observe directly. Traditionally, the standard clonal evolutionary model, based on Darwinian principles and linear progression, was proposed to explain cancer development, especially for chronic myeloid leukemia [5]. However, this linear model struggles to explain most solid tumors. Recent single-cell sequencing data have rejected the linear stepwise model, leading to the emergence of alternative models such as branching evolution, neutral evolution, and punctuated evolution [6].

However, in my opinion, the most significant phenomenon that the SMT fails to adequately explain is “tumor reversion.” The aim of this essay is, therefore, to investigate how the epistemological and ontological tools needed to model tumor reversion processes may reshape our perspective on cancer and offer new heuristic models for cancer research. Rethinking the carcinogenic model is mandatory to plan a very different strategy in cancer treatment.

## TUMOR REVERSION

New evidence suggests that the tumor microenvironment plays a pivotal role in modulating cancer progression. Empirical data from experimental models demonstrate that cancer cells can revert to normal phenotypes when exposed to specific microenvironments, underscoring the limitations of reductionist gene-centric theories [7]. The first clinical evidence of spontaneous cancer regression came from teratocarcinoma. In 1907, the Swiss pathologist Max Askanazy observed the spontaneous reversion of an ovarian teratocarcinoma [8]. A more thorough study of these processes was possible in 1954, after Stevens and Little’s work on the 129/SvJ mouse led to a model of teratocarcinoma [9]. In 1959, Pierce made his first observations on the spontaneous differentiation of embryonal cancer cells derived from testicular teratocarcinoma in the 129/SvJ mouse [10]. Pierce highlighted the pivotal role of the cell microenvironment and introduced the hypothesis of developing methods to direct the differentiation of embryonal carcinoma cells to benign forms as a logical means of controlling this type of cancer [11]. These results were instrumental in introducing the concept of “tumor reversion,” indicating the recovery of a normal phenotype by cancerous cells when exposed to a specific microenvironment.

In 1974, Brinster confirmed Pierce’s hypothesis by injecting testicular teratocarcinoma cells from the 129/SvJ black agouti into murine blastocysts, which were then implanted into albino female mice. This resulted in healthy black-white hybrid offspring, suggesting that “the embryo environment can bring under control the autonomous proliferation of the teratocarcinoma cells [12].” Similar results were obtained by Mintz and Illmensee, who injected embryonal cancer cells into 280 different blastocysts that were further implanted into adoptive mothers. Both fetal and offspring analyses showed no signs of cancer cells. Further tests, such as those analyzing the hair composition, circulating blood cells, and the protein composition of urine, indicated that the teratocarcinoma cells integrated into normal tissue development, forming “mosaics [13].”

Recent studies suggest that while gene-centered Darwinian principles have been shown to explain tumor evolutionary trajectories in many cases, additional evolutionary concepts beyond Darwin’s ideas are necessary to reconcile the full spectrum of evolutionary behaviors in cancer. In particular, increasing evidence now supports macroevolutionary leaps as a feature of cancer, which are likely interspersed with phases of microevolutionary gradualism. Furthermore, evidence of discordant inheritance patterns between cells, neutral evolution, cellular plasticity, and the role of the tumor microenvironment all necessitate the consideration of a broader set of evolutionary models. Understanding how tumor evolution impacts disease progression, and how these processes are shaped by environmental factors and treatment, is critical [6].

Subsequent experiments demonstrated the role of the embryonic microenvironment in controlling the proliferation of different cancer types such as Rous sarcoma, leukemia, neuroblastoma, melanoma, colon, and breast cancers, to mention a few [7]. Despite this experimental evidence, the “reversion” approach in cancer research remains insufficiently explored. One reason may be the lack of epistemological and ontological tools needed to model reversion processes.

## REFRAMING CANCER: FROM MACHINE TO INFORMATION-PROCESSING SYSTEM

The reframing of cancer as an information-processing system necessitates a shift from reductionist metaphors. By embracing process-oriented perspectives, researchers can explore dynamic cellular interactions and adaptive behaviors.

Metaphors involve referring to one thing by the name of another, creating a deliberate category mistake that both the speaker and listener understand. In both science and philosophy, contradictions are typically resolved, but metaphors allow us to introduce new meanings and expand understanding using existing language. By describing the unknown through the known, metaphors help break the limits of current understanding without requiring a new language. However, metaphors can create problems. As they expand, their inherent contradictions become more apparent. An example is Newtonian mechanics, initially viewed as universally true but later challenged by Einstein's theory of relativity. Over time, metaphors can become dogmatic, limiting understanding instead of expanding it [14].

Machine metaphors abound in life sciences: animals as automata, mitochondria as engines, brains as computers. In mechanistic thought, systems like machines follow a fixed set of rules with clear distinctions between intrinsic (internal) and extrinsic (external) factors. However, organisms operate differently. The dynamic structures of living beings, which depend on the interaction between internal and external factors, are far more complex than machines [15]. Processes like tumor reversion make the situation of organisms even more complex. This process is akin to a broken machine repairing itself. We can liken this situation to a car with a broken axle on a rough road, where the axle repairs itself once the car reaches a smooth road.

A key feature of organisms is their autonomy in their relationship with the environment. While an organism determines its interactions with the environment through its dynamic processes, machines are driven by external factors. The organism’s sensitivity to its environment is its “Umwelt,” or its own unique world. This shows that organisms have the ability to create their own environment and develop internal relationships in this process [16].

Organisms process information to sustain their existence. This process extends from molecular structures to ecosystems, with increasingly complex systems emerging at each level. Information processing occurs as organisms filter and synthesize data from their environment. This process is called functional information and plays a critical role in the functioning of biological systems. Functional information is a key factor in organisms’ ability to organize themselves and form complex structures. Life can be viewed as units that process information at every level. At the molecular level, cells function as information-processing systems within organisms, while ecosystems operate as large-scale systems managing the flow of information between organisms. This information processing includes the organisms’ ability to self-regulate, known as autopoiesis (self-renewal), and their capacity to adapt to their environment [17, 18].

## CANCER AS A CHAOTIC INFORMATION-PROCESSING SYSTEM

A novel perspective frames cancer as a complex information-processing system, rather than solely a genetic disorder. It draws parallels between biological systems and computational frameworks, suggesting that cellular life, particularly in the context of cancer, operates as a hierarchical network of information processing. Stem cells are central to this discussion, viewed as adaptable “information processors” capable of self-renewal and differentiation. The heterogeneity and plasticity of stem cells, especially in response to environmental stress, reveal the intricate balance of intrinsic and extrinsic factors that regulate their function. Chaotic gene expression, traditionally seen as detrimental, is reinterpreted as a mechanism that fosters cellular flexibility and adaptability in hostile environments. The study delves into how chaotic feedback loops within stem cell populations and their niches can drive carcinogenesis, emphasizing the role of both positive and negative feedback in shaping cellular evolution. By examining the trial-and-error dynamics of chaotic systems, the essay underscores the importance of chaos in evolutionary processes, where adaptability and innovation arise from the complex interplay of stability and instability. This perspective challenges the reductionist, gene-centric model of cancer and advocates for a holistic understanding of the disease as an emergent phenomenon shaped by multifaceted information dynamics [19].

## CONCLUSION

Viewing cancer specifically, and biological systems in general, as machines fails to explain certain phenomena, particularly tumor reversion. I believe that seeing biological systems/tumors as information-processing systems will open new horizons. However, this is not easy—it requires adopting a process ontology instead of an atomistic-materialistic one. The ability of researchers to break free from dogmatic perspectives and develop the capacity to view phenomena differently would be a significant gain for science.
